# Investigative and extrapolative role of microRNAs’ genetic expression in breast carcinoma

**DOI:** 10.12669/pjms.323.9321

**Published:** 2016

**Authors:** Ambreen Usmani, Amir Ali Shoro, Bushra Shirazi, Zahida Memon

**Affiliations:** 1 Prof. Ambreen Usmani, Prof. & HOD, Anatomy, Bahria University Medical and Dental College, Karachi, Pakistan; 2 Prof. Amir Ali Shoro, Principal & Dean, Professor of Anatomy, Liaquat National Hospital & Medical College, Karachi, Pakistan; 3 Prof. Bushra Shirazi, Professor of Surgery, Associate Dean-Clinical, Ziauddin University, Karachi, Pakistan; 4 Prof. Zahida Memon, Professor & HOD-Pharmacology, Associate Dean-Pre Clinical, Ziauddin University, Karachi, Pakistan

**Keywords:** miRs, Breast carcinoma

## Abstract

MicroRNAs (miRs) are non-coding ribonucleic acids consisting of about 18-22 nucleotide bases. Expression of several miRs can be altered in breast carcinomas in comparison to healthy breast tissue, or between various subtypes of breast cancer.

These are regulated as either oncogene or tumor suppressors, this shows that their expression is misrepresented in cancers. Some miRs are specifically associated with breast cancer and are affected by cancer-restricted signaling pathways e.g. downstream of estrogen receptor-α or HER2/neu.

Connection of multiple miRs with breast cancer, and the fact that most of these post transcript structures may transform complex functional networks of mRNAs, identify them as potential investigative, extrapolative and predictive tumor markers, as well as possible targets for treatment. Investigative tools that are currently available are RNA-based molecular techniques. An additional advantage related to miRs in oncology is that they are remarkably stable and are notably detectable in serum and plasma.

Literature search was performed by using database of PubMed, the keywords used were microRNA (52 searches) AND breast cancer (169 searches). PERN was used by database of Bahria University, this included literature and articles from international sources; 2 articles from Pakistan on this topic were consulted (one in international journal and one in a local journal). Of these, 49 articles were shortlisted which discussed relation of microRNA genetic expression in breast cancer. These articles were consulted for this review.

## INTRODUCTION

In this review article we have focused on the capability of miRs to act as diagnostic and prognostic biomarkers. We outlined their prospective ability to correlate with breast cancer and will abridge their ability to give information on its prognostic value. We have also focused on miRs validated by various researches.^1^

## CHARACTERISTICS OF MICRORNA

The characteristic features of miRs are as follows:

- Post-transcript RNAs
- Non-coding RNA molecules of ~22 nucleotides
- Present as single units or in gene clusters
- Possess a seed sequence, a sequence composed of nucleotides 2 through 8 of its 5’ end
- “Influence the stability and translational efficiency of target mRNAs”

MicroRNAs are born by a series of actions; these actions occur first in the nucleus and then in the cytoplasm. In the nucleus they are predominantly transcribed as primary-mi-RNA (pri-miRNA) under the action of RNase polymerase II which is ~ 100-1000 nucleotide in length.^1^ This is followed by the process of capping and polyadenylation. Further this pri-miRNA is cut by RNase III, DROSHA and its co-factor DGCR8 into smaller ~ 70 nucleotide stem loops called as pre-RNA.^2^,^3^ The pre-RNA moves from the nucleus to the cytoplasm by means of exportin-5. The loop region of pre-RNA is removed by DICER (RNase III) and its binding partner TRBP. A mature miRNA-miRNA* duplex is released, the single dominant strand is incorporated into RISC (RNA induced silencing complex) to gradually regulate gene expression by complementary-base pair interaction ensuing in interference with translational ability and stability of target mRNA or it may result in its degradation.

The objective of this review was to correlate the diagnostic, prognostic and therapeutic value of miRs with breast cancer in women as validated in several studies.

## ASSOCIATION OF MICRORNA WITH BREAST CANCER

**Fig.1:**
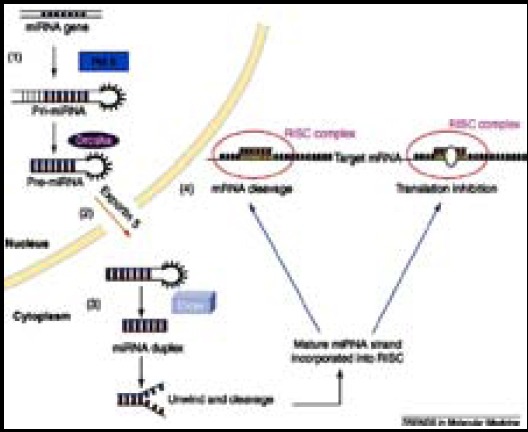
Source: Garzon R et al. MicroRNA expression and function. Trends in molecular medicine 2006

**Fig.2:**
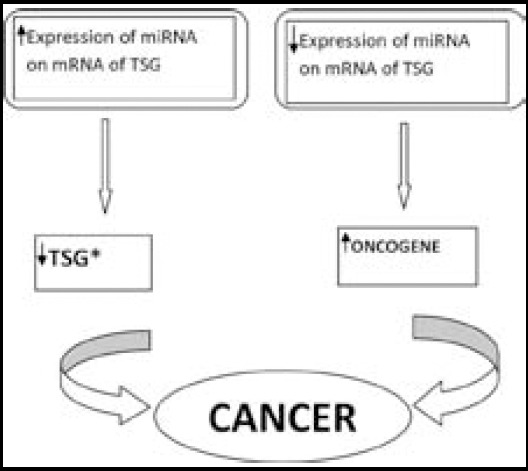
Relation between expression of micro RNA and cancer *TSG- Tumor suppressor gene.

The association of multiple miRs with breast cancer, and the fact that most of these miRs may amend complex functional networks of mRNAs, identify them as potential investigative, extrapolative and predictive tumor markers, as well as possible remedy targets (Fig.1).^1^ An additional advantage of miRs in oncology is that they are extraordinarily stable, secure and are detectable in serum, plasma, urine, saliva and tissue. MicroRNAs function as oncogenes or tumor suppressor genes. (Fig.2) Breast cancer biomarkers include: CA-125, CA 15-3 (“carbohydrate antigen 15-3”), CEA (carcinoembryogenic antigen) and “TPS (tissue polypeptide specific antigen)”. But the usual biomarkers presently used for evaluating breast cancer are ER-α and HER2/neu.^2^

While breast cancer subtypes have been defined by mRNA expression profiling, additional molecular foundation of these subtypes can be portrayed by miRNA expression profiling. Accurate subgroups of breast cancer can be acknowledged with these targets that can subsequently be used for treatment.^3^ Explicit part of miR biogenesis that can predict their potential as biomarkers is their noteworthy stability in formalin which is used for tissue fixation as well as their stability in plasma, serum and other body fluids.^4^

As these miRs are preserved in a stable state in serum and plasma, sheltered from RNase activity their expression patterns can be discovered from these fluids without resorting to an invasive procedure like biopsy.^5^,^6^ A study shows that miR expression patterns were evaluated from human sera in five different types of human cancers which included ovarian, colon, prostrate, lung and breast cancer. The assessment was done using miR high density microarray and ample amount of miR were detected in one ml of serum to discover miR expression configurations without the requirement for amplification procedures. These miR expression configurations were used to differentiate between normal and cancer patient samples.^7^-^10^ Several miRs are shown in Table-I with their mode of regulatory expression.

**Table-I:** Expression status of microRNA in breast cancer^1^,^2^,^8^,^18^,^25^,^32^,^36^,^22^,^40^

*Expression in breast cancer*	*MICRORNAs*
Upregulated	miR-7 and miR-128 (aggressive tumors), Let-7, miR-21, miR-27a, miR-29a/b-1/b-2/c, miR-34, miR-98, miR-122, miR-155, miR-181b-1, miR-182, miR206, miR-210 (triple negative), miR-213, miR-221, miR-365, miR-516-3p, miR-520
Downregulated	Let-7a, miR-9-1, miR-10b, miR-17-5p, miR-20b, miR-27b, miR-125a/b, miR-126, miR-130a, miR-140, miR-145, miR-146, miR-205, miR-206
Highly expressed	miR-9-1, miR-10b, miR-21, miR-25, miR-93, miR-106b, miR-222
Increased expression in ER positive	Let-7b/c/f, miR-7, miR-10a, miR-29a/b-1/b-2, miR-128, miR-130, miR-224. Including PR positive: miR-24, miR26a/b, miR-29c, miR-30a-3p/30a-5p/30b/c/d
Multidrug resistance	miR-145, miR-210, miR-221, miR-222, miR-421
Aberrant hypermethylation	miR-9-1, miR-34b/c, miR-124, miR-148, miR-152, miR-663
Migration, invasion and metastasis(Potential targets*)	miR-125a(HER2/3, HuR), miR-125b (HER2/3, c-Raf), miR-200 (BMI1, ZEB1/2, PLCG1), let-7 (LIN28, HMGA2), miR-30a(vimentin), miR-7 (Pak 1), miR-126(SOX4, TNC), miR-10b (HOXD10), miR-21(BCL-2, TPM1, PDCD4, PTEN, MASPIN), miR-155 (Rho A, SOCSI), miR-221 and miR-222(TRPS1), miR373 and miR-520c (CD44), miR-182 and miR-183 (CBS7, DOK4, NMT2, EGR1)

* HER2/3=human epidermal growth factor2/3, HuR= RNA binding protein that stabilizes mRNA, c-Raf= cellular rapidly accelerated fibrosarcoma, BMI1= B lymphoma Mo-MLV insertion region 1homolog, ZEB1/2= zinc finger E-box binding homeobox 2, PLCG1= phospholipase C gamma 1, LIN28= lin28 homolog A (C.elegans), HMGA2= high mobility group AT-hook 2, Pak=p21-activated kinase 1, SOX4= sex determining region-box 2, TNC=tenacin, HOXD10=homeobox D10, BCL=antiapoptoticbcl, TPM1= tumor suppressor tropomyosin 1, PDCD4= programmed cell death-4, PTEN=phosphatase and tensin homolog, Rho A= Ras homolog gene family member A, SOCSI= suppressor of cytokine signaling 1, TRPS1=tricho-rhino-phalangeal syndrome type 1 protein, CD44= cell surface protein, CBS7=cystathionine β-synthase, DOK4= downstream of kinase-4, NMT2= native metallothionein, EGR1=early growth response gene.

## METHODS FOR DETECTING MICRORNA EXPRESSION

Several methods can be used to detect and identify the presence of miRs, some are conventional methods and others are newer. Once a standardized method has been devised it will be easier to assess lower concentration of miRs which may not be measurable by conventional procedures. In order to improve more “sensitive detection methods”, it is imperious that the markers which measure the miR levels have a low recognition limit. Each technical method has its own benefits and disadvantages. The numbers of methods for miR analysis are swiftly increasing but most of these methods continue to entail standardization. These standardized methods must be dependable for diagnosis and should also be reproducible, precise and robust. The current molecular investigative tests show potential in augmenting the regular methods of evaluating cancerous “status and treatment” decisions for patients with breast cancer. However research must also establish normal levels of miR to define whether the miR is up regulated or down regulated. A noteworthy disadvantage to miR study is the existing lack of a reliable, universal control. In case a normalizer is proven, miRs are likely to be understood by physicians to support patient care. The chances of commercial availability of miRs assay within the next few years are realistic provided it is cost effective. Various methods can be used for identifying the presence of miRs but they all have their pros and cons. The methods that can be used include:^8^,^9^

**a). *Northern Blotting:*** The earliest technique used for detection of RNA for the study of Gene expression was Northern Blotting. The technique involves the use of electrophoresis to separate RNA samples by size and to detect the target RNA with a hybridization probe complementary to the target RNA sequence.^10^ The probes are composed of nucleic acids having a complementary sequence to all or part of RNA to be isolated. The term ’northern blot’ actually refers specifically to the capillary transfer of RNA from the electrophoresis gel to the blotting membrane. Locked nucleic acid (LNA) or modified oligonucleotide probes can be used for “in situ” detection of miR.^11^,^12^

The disadvantage of this technique is that at first extraction of total RNA from a homogenized tissue sample or from cells is required and then the required RNA such as miR can be isolated. It is a semi quantitative procedure and also time consuming therefore cannot be used for routine diagnostic and/or prognostic purpose.

**b). *Cloning:*** This newer technique is mostly used to discover novel miRs but it is not a precise approach for their quantification. Many procedures have been used for miR cloning but the popular one is miR serial analysis of gene expression (miR age).^13^-^15^

**c). *Real Time PCR:*** This is a solution phase method used mostly for quantification of specific miRs. Several specific quantitative Real Time-Polymerase Chain Reaction (qRT-PCR) procedures have been developed and optimized for miR detection. Methods (Real Time) built upon this “reaction with stem loop primer followed by a TaqMan PCR analysis is an end point and real time looped RT-PCR procedure”. This process is a high sensitive procedure as PCR amplification is precise and correct. However due to its cost it is impractical to adapt this tool routinely for patients.^16^ Asaga et al.^17^ demonstrated the potential utility of the novel “reverse-transcriptase quantitative real-time PCR (RT^2^-qPCR)” to identify and measure the concentrations of circulating miR-21 from plasma and sera in patients with malignant breast carcinoma.

**d). *In SITU Hybridization(ISH):*** It is a type of hybridization that uses a labelled DNA, RNA or a modifies nucleic acid strand (i.e. probe) to localize a specific DNA or RNA sequence in portion or section of tissue (i.e. in situ). This method has the ability to provide evidence on the site of miRs presence in cells or tissue.^18^

**e). *Microarrays:*** This is presently the most frequently used method to detect miR levels. A microarray is a multiplex lab-on-a-chip. It is a 2D array on a solid substrate such as a glass slide that assays large amounts of biological material using throughput screening, processing and detection methods.

This technology uses miR oligonucleotide probes amine-modified 5’ termini, which are mobilized onto miR microarray made on glass slides. Detection of miRs in serum samples and the ability to differentiate between normal and breast cancer has been established by this method.

This technique can analyze hundreds of sample in short time but its high cost is the main obstruction for its utility as a routine test.^19^

**f). *Electrochemical Detection:*** This method is also a solid phase miR analysis technique which is related to microarray analysis. For this procedure to take place target molecules are directly conjugated to redox active and electrocatalytic moieties through coordinative bonds with purine bases in the miR molecule.^20^ A novel electrochemical miR discovery process based on “enzyme amplified biosensing” of miR-21 from cell lysate of total RNA was established by Kilic et al.^21^ In this method total RNA encompassing miR was secluded from breast cancer cell lysates and “oxidation signal of the enzymatic reaction product, alpha naphtol” was measured. After the entire procedure a picomole range of exposure was reached as biomarker for miR. Another study by Labib et al.^22^ have developed “three- mode electrochemical sensor” for revealing and quantization of extremely low amounts of miRs in an extensive vibrant range of calculated absorptions. The sensor enables three detection functions founded on hybridization, p 19 protein binding and protein dislodgment. Together the three mode sensor shows as low as “5 aM or 90 molecules of miR per 30 µL” of sample without PCR augmentation and can be controlled in an active variety from 10aM to 1µM.

**g). *Fluorescence Correlation Spectroscopy:*** It is an analysis of the concentration fluctuations of fluorescent particles (molecules) in solution. The target nucleic acid is probed with a tagged fluorophore and the fluorescence emitted from this complex is observed.

This technique is a solution-phase procedure based on two-colour concurrent detection in which “two organic fluorophore-labeled oligonucleotides” are pointed to miR targets. These two spectrally distinguishable organic fluorophore-labeled oligonucleotide probes hybridize with their target nucleic acid which in turn can be quantified by a detection instrument. Two discrete fluorophores are considered and the quantity of molecules available from these counts is measured “using an algorithm”. The maximum value of quantization for this technique has been stated as 500fmol. Its extraordinary sensitivity, the quickness of the assay and its capability to state the difference between single base mismatch are the advantages of this method. However it is not free of disadvantages being the prerequisite of an exterior excitation basis to stimulate the fluorophores resulting in lessening the assay’s sensitivity.^12^,^23^

**h). *Surface-Enhanced Raman Spectroscopy (SERS):*** Driskell et al.^24^ described this solid phase method in which “silver nanorods are adsorbed” on top of a glass slide and then miRs are mixed with the nanorods. After the miRs are attached to the nanorods, “surface enhanced Raman scattering spectra” are used. As a result heights (peaks) are formed which are then utilized to recognize miRs. Also, this does not need a label for recognition. A disadvantage of the given methodology is related to the spectra, which must be taken separately earlier to performing the assay to recognize their characteristic Raman shift.

## METHODOLOGY

Literature search was performed by using database of PubMed, the keywords used were microRNA (52 searches) AND breast cancer (169 searches). PERN was used by database of Bahria University, this included literature and articles from international sources; two articles from Pakistan on this topic were consulted (one in international journal and one in a local journal). Of these, 49 articles were shortlisted which discussed relation of microRNA genetic expression in breast cancer. These articles were consulted for this review.

## DISCUSSION

As developing confirmations point the significance of miRs in diagnosis and prognosis, the usefulness of miR based breast cancer therapy is also being explored. Several miRs have some association with cancers, these are thought to control process of cell cycle and developmental processes.^25^,^26^ The therapeutic strategies based on miRs suggest a substitute for “targeting multiple gene networks” that are under the control of a single miR. These approaches can be expressed by either antagonizing or reinstating the functions of miRs^27^,^28^ “Anti-miR 2-O-methyl” or “blocked nucleic acid oligonucleotides” can be used to incapacitate oncogenes. Pharmaceutical companies are working on the production of anti-molecules which may remove the breast cancers cells resulting in prevention of the type of cancer and also may reduce tumor growth. Anti-miR-21 induced decrease in cancer proliferation was exposed by the introduction of topotecan which is a chemotherapeutic drug, this is an inhibitor of “DNA topoisomerase I”.^29^ Such novel events highly recommends that clampdown of the oncogenic miR-21 could alert or sensitize tumor cells to anti-cancer therapy. However this is a promising outlook for patients displaying a poor response to initial stages of chemotherapy.^3^ Another point of interest shows the capability of miR 34a to “inhibit proliferation and migration of breast cancer through downregulation of Bcl-2 and SIRT1”.^30^

On the other hand, the induction of tumor suppressor miR expression using “viral or liposomal delivery of tissue-specific tumor suppressors” to the wounded tissue may result in the prevention of tumor aggression or may cause shrinkage of the breast tumor. For instance miR-145 appears as one of the most consistently down-regulated miR in breast cancer when compared to normal tissue. In addition, miR-145 may be detected in serum samples from cancer and normal patients^31^ miR-145 re-expression in breast cancer was accompanied by a “pro-apoptotic effect, dependent on TP53 activation”, while TP 53 can, in turn provoke miR-145 expression, thus forming a death promoting link among miR-145 and TP53. Moreover miR-145 can down regulate ER-α protein expression through contact with two complimentary locations inside its coding sequence. This supports an opinion that miR-145 re-expression remedy could be planned in certain group of subjects with Estrogen Receptor-α-positive and/or “TP53 wild-type tumors”.^32^

As reported by several laboratories other target miRs may be associated to more specific pathways, such as the HER2/neu family mediated or Estrogen Receptor-α-driven motioning. Examples of such miRs are “miR-18a, miR-22, miR-181, miR-206, miR-221/222” that have proven to be a part in downregulation of Estrogen Receptor-α expression and in suppression of ER-α-mediated motioning in breast carcinoma cell lines.^33^ By adapting the signatures of these miRs, it is probable to find a novel way of remedy for ER-α-negative breast cancer. Researchers show another finding indicating that miR-205 is able to interfere with the proliferative pathway mediated by ERBB receptor family which also includes HER2/neu, and to increase the responsiveness of breast cancer cell lines to “tyrosine-kinase inhibitors gefitinib and lapatinib”. This suggests that the restoration of miR-205 could improve the responsiveness of breast cancer cell to specific anticancer therapies.^8^

Scott et al. explained that infection of breast cancer cell lines with retroviral concepts showing either miR-125a or miR-125b resulted in clampdown of HER2/neu.^33^ Such miR could be targets to explore the mode of action of HER2/neu inhibitors for example trastuzumab.^34^ Studies show that miR-155 is expressed in different ways in sera of women with progesterone positive tumors as compared to others.^30^ Other miRs of interest include miR-200 family members and miR-205, which appear to exert a significant role in the control of epithelial-mesenchymal transition.^32^ Usmani et al.^35^ investigated miR-21 in women suffering with invasive ductal carcinoma of stage III, this showed high expression of this microRNA when compared with healthy individuals. However it was also shown that the daughters of these cases also expressed miR-21 which was significantly higher than in healthy individuals but less expressed when compared with their mother with stage III invasive ductal carcinoma.^35^ This transition is considered as an important and necessary step to help the breast cancer cell lines to escape from the primary tumor; the mechanism of this process has not yet been clearly defined Approaches based on miR could help to counter the metastatic process which is still incurable in breast cancer.

Several researches have indicated that multidrug resistance can result as a barrier for treatment of breast cancer. Chemotherapy quite frequently fails to attain the successful treatment results; this can be attributed to resistance of the drugs to work at its full potential. Such resistant cases can be often interceded by drug efflux carriers for example P-glycoprotein, these are often overexpressed in cancer;^36^ miRs have been appraised for their significance in foreseeing reaction to hormonal cures and chemotherapies. miR- 451 is an example that discovered to be connected with multidrug-resistance genes in MCF-7 cells^37^ Scientists have pointed out that “doxorubicin (DOX)- resistant breast cancer cell lines” revealed modifications in miR profile and miR-451 was recognized to “regulate the expression of multidrug resistance gene”. It was further shown that reinstating miR-451 into “DOX-resistant breast cancer cell lines” culminated in amplified sensitive reaction of cells to DOX. This indicated that reinstatement of such transformed miR expression may have noteworthy effects for breast carcinoma cure. Tamoxifen-resistant cells show an upregulation of miR-221/222 as compared to tamoxifen-sensitive cells.^38^,^39^

Liang et al. explained in detail some of the miRs that are involved in drug resistance, which include miR-326,^40^ miR-328 and miR-34a.^39^ It is determined that miR-31a is a straight target of p53,^31^ “miR-21 overexpression is associated with the development and progression of multidrug resistance in breast cancer” and hopefully also rising as an original and optimistic reversing target.^31^,^40^

Lacroix et al. describes that as evolving signs magnify the worth of miRs in diagnosis and prognosis of breast cancer there is still an increasing necessity for supplementary dependable “molecular markers” as the perfect indicator of disease. The ideal investigative marker should be specific and sensitive. It should also be easily measureable in a reproducible way through simple, fast and economical techniques.^32^ Due to the complexity of breast tumors more robust markers must be identified.^40^ It is likely that miR signatures which are at present showing ability of precisely categorizing tumors in accordance to accessible prognostic variables, will aid as new biomarkers and extrapolative indicators. Research indicates the accuracy of derived “genomic signatures” of miRs to have the potential to advance greatly. While breast cancer subtypes have been defined by mRNA expression profiling, however, complete subclasses of breast cancer via “miR expression profiling” could be further characterized on the basis of molecular research. This could possibly outline accurate subcategories of breast carcinoma, and afford chances for the documentation of “novel targets” that may be explored further.^2^,^4^,^30^

In breast cancer target miRs can be alleged oncogenes or tumor suppressor genes also found in various other cancers (Table-I) these include both circulating and non-circulating miRs. Most targets of these miRs appear to be linked to cell proliferation and migration^16^ this has shown linkage of miR with aberrant hyper methylation, the author showed this for “miR-9-1, miR-124, miR-148, miR-152 and miR- 663 in 34-86% of cases in a series of 71 primary human breast cancer specimens”. In addition miR-9, miR-34b/c along with miR-148a remained discovered to experience explicit “hyper methylation-associated silencing” in tumorous cells as matched to normal tissue. Hyper methylation of the given miRs was also seen in 207 patients in a study by Kovalchuk O.^37^ It has been noted that early exposure to xenoestrogens is riskily and incline to breast cancer in later life. It is expected that longer lived, self-forming epithelial progenitor cells are more susceptible to these injuries and convey the incapacitated recollection through epigenetic changes to their differentiated descendants. Functional analysis of miR-9-3 is involved via the p53-related apoptotic pathway, it is therefore likely that the “epigenetic silencing” of miR may weaken cellular mission resulting in the increase of breast cancer cell lines.^38^ These miRs show abundant potential as new diagnostic and prognostic markers in breast cancer. However miR assays do not presently recognize control, against which they can be compared. Research has yet to demonstrate ease in reproducibility.^39^

MicroRNA’s dependability as a screening tool has not yet been recognized extensively. However since miR species can be discovered in archived sera from breast cancer patients it may be hypothetically possible.^31^,^38^,^40^ The proof that serum/plasma analysis could precisely identify cancer cells without an invasive procedure which brands miRs potentially suitable for cancer screening in populations of high threat. These small post-transcripts show promise in foreseeing response to hormonal treatment including chemotherapies, in that way they can become “unique predictive factors” in breast carcinoma.

## CONCLUSION

The memory of multiple miRs with breast cancer, and the fact that most of these post transcript molecules may curb complex functional complexes of mRNAs, identify them as potential investigative, prognostic and predictive tumor markers, as well as possible therapeutic targets is still under experimental phase. The association between transmuted miR discovery and breast cancer discovery including metastasis is either via the defeat of tumor suppressor or the overexpression of oncogenic miRs. Several techniques have been identified for indicating the presence of miRs which have been highlighted in this article. An additional advantage of miRs in oncology is that they are remarkably stable and are notably detectable in tissue, serum, plasma, urine, saliva and other body fluids. Therefore, enhanced knowledge of these genes controlled by certain miRs may support the complete prospective of miRs in regards to cancer diagnosis, prognosis and treatment. This is a promising breakthrough and will help in cancer therapy after proper identification of the troublesome miRs.
